# Measuring gender attitudes: Developing and testing Implicit Association Tests for adolescents in India

**DOI:** 10.1371/journal.pone.0264077

**Published:** 2022-06-16

**Authors:** Tarun Jain, Diva Dhar, Vrinda Kapoor, Vrinda Kapur, Anita Raj

**Affiliations:** 1 Indian Institute of Management Ahmedabad, Ahmedabad, India; 2 University of Oxford, Oxford, United Kingdom; 3 Cornell University, Ithaca, New York, United States of America; 4 Porticus, New Delhi, India; 5 University of California San Diego, San Diego, California, United States of America; Bucharest University of Economic Studies, ROMANIA

## Abstract

We develop and test gender attitude measures conducted with a school-based sample of adolescents aged 14–17 years in India. We test a measure with survey items and vignettes to capture gender-based value and stereotypes, an Implicit Association Test (IAT) capturing gender-based value, and an IAT capturing gender stereotype. All demonstrate good internal reliability, and both IATs are significantly associated with our survey measure suggesting criterion validity, though not confirming it due to the lack of a gold standard measure on gender attitudes. Finally, construct validity is indicated from the measures’ positive significant associations with higher girls’ mobility and education. The gender-related IAT tools developed are consistent and valid, and modestly correlated with gender-related behavior outcomes such as mobility and school enrolment.

## 1. Introduction

A growing body of research from different settings demonstrates that gender norms and roles—the range of socially constructed behaviors and attitudes expected and even required for people based on their sex—harm the health and well-being of females, males, and those outside of the gender binary [1]. Among women, for example, these norms can restrict employment opportunity and disproportionately burden them with household responsibilities, affecting reproductive autonomy and health care access. Norms may encourage men to behave harmfully through greater substance use or unsafe occupations (e.g., workers in the mining industry in developing countries often fall under the informal sector where labor and safety laws are either non-existent or lax) that increase their risk for pre-mature mortality. Research demonstrates that constraining people within public and private spheres of life due to norms and roles not only harms individuals, but also compromises economic development, political governance, and climate action at a national scale [2–4]. A growing literature studies attitudes and gender norm change in developing countries. Bandiera et al. (2020) study the effect of livelihood training on female empowerment in Uganda, and Ashraf et al. (2020) study negotiation skills training for girls in Zambia [5, 6]. Consequently, there is increasing interest in altering gender norms to improve health and society, with a focus on youth as a population amenable to change [7]. Further, while gender socialization (environmental training on these norms) starts at birth, early adolescence is when gender attitudes take root and is thus important in understanding and affecting gender attitudes [7]. Our paper focusses on measuring these gender attitudes, gender-based behavior and norm change amongst adolescents, which could help policymakers devise better gender equal policies and help erode centuries-old regressive gender and cultural norms.

Research on gender attitudes among adolescents in low and middle-income countries (LMICs) is nascent despite recognition for more focus on these issues in contexts such as South Asia [8]. Qualitative and quantitative research on early adolescents in India highlights that gender socialization, largely transmitted to youth by parents, restricts mobility, dress, and interaction with the opposite sex more for girls relative to boys, with greater negative consequences for girls who do not adhere to gender norms [9, 10]. Basu et al. (2017) also found fewer expectations and support related to education and employment for girls relative to boys, even at this early age [9]. These findings highlight differences in attitudes of acceptability toward certain behaviors in ways that over-restrict girls and may foster unhealthy behaviors (e.g., substance use, physical risk taking) among boys as a means of showing masculinity. Evidence from the region suggests that school-based interventions focused on gender norm changes can have positive impact on norms and school attendance [11–13].

While these findings demonstrate the importance of measuring and understanding these issues, quantitative measures are currently limited by the lack of standard measures on these attitudes and contextual considerations for India. Prior reviews of the literature found that much of the gender measures development comes from North America and Western Europe and focuses on adults and older adolescents rather than early adolescents [7, 14]. The only attitudinal survey measure developed for use with early adolescents in LMICs including India was limited to attitudes toward romantic engagement, inclusive of sexual expectations and the double standard (acceptability and even social value for males dating, where female dating is viewed unfavorably [15]). Attitudes toward gender stereotypes beyond dating exist for adults, though they are largely self-reported survey items [16–19], and are thus more vulnerable to social desirability bias and falsification [20, 21]. These findings point to the need for tools to measure more general gender attitudes among early adolescents residing in LMICs.

Novel behavioral science methodologies able to capture gender attitudes but less vulnerable to bias include vignettes and Implicit Association Tests (IAT). Vignettes involve text or stories to which participants can respond regarding their thoughts on a topic, or what they think others might think or do related to the topic or circumstance [22]. Researchers use this approach to measure participant attitudes of acceptability of behaviors as well as perceptions of potential social sanctioning against the behaviors, based on sex of the individual in the story and sex of the respondent [23–25]. A more objective rather than subjective means of assessing the attitudes (e.g., asking about what others believe or what is likely to happen in the story) offers an approach that is less vulnerable to social desirability bias. IAT is a computer-based test in which participants must rapidly categorize two target concepts (e.g., “male” and “female”) with a characteristic (e.g., “teacher” or “construction worker”) [26]. IAT measures both connection of a given concept with the characteristic, as well as the time required by the participant to make that connection, allowing assessment of both self-reported attitudes and comfort with that attitude based on the relative time required by the participant to make that connection [26, 27]. By capturing response times between these attributes, the IAT is able to consider discomfort and possibly social desirability in responses [26, 27]. Researchers have only more recently started using vignettes to assess gender attitudes among early adolescents in LMICs but show success, at least in terms of gendered attitudes toward romantic partnering [28]. Researchers have used IATs [29–31], including measurement of gender attitudes related to women in politics in India [32], but no published studies have assessed gender attitudes with early adolescents, in India or elsewhere.

This study involves psychometric testing of gender attitude measures for early adolescents in India for gender-based value (preference of boys/men over girls/women indicated by positive/negative attributes, prioritization for opportunities and resources) and gender stereotypes (attitudinal beliefs regarding females relative to males on types of employment and domestic responsibilities). We include three new measures for consideration: 1) an IAT capturing gender-based value—a “taste-based” IAT, 2) an IAT capturing gender stereotypes, and 3) a survey measure inclusive of both survey items and vignettes to capture gender-based value and stereotypes. We developed these measures for inclusion at follow-up for an evaluation of an intervention designed to support more equitable gender attitudes among middle school students in India. Findings from this work offer novel gender attitude measures that can be applied for monitoring and evaluation of these attitudes in early adolescents in India, a nation with over 250 million adolescents, as well as for adaptation and use in other LMIC settings.

## 2. Study design

We analyzed data from 6458 adolescents aged 14 to 17 years who participated in the three-year follow-up survey conducted for a two-arm cluster randomized trial evaluating a year-long gender attitude change program for middle school students in four districts of Haryana, India (Sonipat, Panipat, Rohtak and Jhajjar). Government schools within these districts (N = 314 schools) were randomly assigned, stratified by district, co-ed status of the school, school size, and distance to the district headquarters, to the intervention condition (i.e., the gender attitude-change intervention; N = 150 schools) or the control condition (i.e., no intervention; N = 164 schools). This sample size was selected to be able to detect statistically significant medium and long-term effects of the program on gender attitudes, behaviors, aspirations, as well as educational and fertility outcomes (Power calculations are available on request.). We recruited and consented approximately 46 randomly selected 6th and 7th graders per school to participate in the evaluation study and followed them again at a three-year follow-up, as 9th and 10th graders in 2016–17. Among these, a subset of 8333 students, i.e., approximately 26 students per school, were randomly selected to respond to the IAT measure at baseline (The research team used the software Inquisit by Millisecond Software to code, administer and collect data from the IATs. The team used the statistical software Stata to analyze the data.). Out of these, 6458 students were retained at follow-up when complete measures of gender attitudes (gender-based values and stereotypes) were administered. We found no significant differences between those retained and lost at follow-up on key demographics including sex (S2 Table in S1 Appendix).

### Procedure

We recruited, consented and conducted behavioral assessments with randomly selected students within each school during the fall of the 2013–14 academic year; follow-up data were collected in 2016–2017. We analyzed follow-up data for the current study, as we only added the full barrage of gender attitudes measures at follow-up.

At study entry, we obtained informed consent both and separately from parents and students prior to surveying students. Male and female research staff members (i.e., enumerators, supervisors and monitors) were hired specifically for this study trained in both data collection and gender equity over a 10-day training period. Sex-matched enumerators approached selected participants who provided verbal or written personal assent and written parental consent (collected prior to data collection) and escorted them to a more private setting for assessment outside the classroom. We replicated this same procedure at follow-up.

Follow-up behavioral assessments, which included the survey and vignette measure as well as the IAT, were approximately 60 minutes in length for most participants, and occurred during or after the school day. Prior to conducting the IATs, a practice round was provided to the students which was focused on matching flowers and insects rather than gender roles. We used this approach to facilitate students’ ability to self-administer the IATs—which is required by the test. Subsequent to the full behavioral assessment, participants were thanked for their time. We provided no incentive payment for participation.

All research protocols and survey instruments received ethics approval from both the Government of Haryana, which formally approved the questionnaires and study protocols and granted us permission to conduct the surveys in schools, as well as the Institutional Review Boards of Northwestern University in the United States and the Institute for Financial Management and Research in India.

### Measures development

In the process of developing the evaluation study in 2013, the research team recognized a lack of measures on gender attitudes and stereotypes developed or validated with adolescents in India and other low and middle-income countries. Consequently, the team made efforts to develop new measures for Indian adolescents based on theory, expert input, prior research and testing on gender attitudes and stereotypes measures, as well as formative research with youth, approaches recommended as standard for measures development [11, 33].

As noted above, IAT development is considerably more complex than measures that can be administered via survey item or vignette format (discussed below). To determine what images, language, characteristics/attributes (‘good’, ‘bad’, stereotypical male and female jobs) should be used for IAT development for Indian adolescents, we obtained input from school teachers, students, and local and national experts on gender attitudes and on adolescent development. The images and language were then constructed and reviewed with these groups again for finalization. Cognitive interviews followed, with students describing their understanding of the images, vocabulary and the tasks in the process. These efforts were all critical in selecting words and images which the respondents could easily understand given their age and environment. For example, the images chosen were those of boys and girls who looked like they belonged to a similar age and background as the respondents. The first IAT measure (IAT1) focused on gender-based value and was developed at the baseline. The second IAT measure (IAT2) focused on gender stereotypes and was only implemented at follow-up. Both IATs were developed in Hindi for administering to the students, and later translated to English for purposes of dissemination. Due to the length of each IAT, we were only able to implement one IAT for a given subject, so we randomly assigned each IAT to a student stratified on their school, grade, and gender, resulting in 3,078 students receiving IAT1 and 3,380 students receiving IAT2 at follow-up (The randomization was done prior to rolling out the survey (stratified on school-grade-gender) so completion rates are different.).

The survey items and vignette measures were developed in parallel with the IAT. Feedback from social science and adolescent development experts helped validate items in terms of face validity for the concepts attempting to be measured, both gender attitudes and stereotypes reflective of the cultural context. The nine-item measure was tested with sixth and seventh graders via cognitive interviews, in which students received the assessment and then explained back to the interviewer what the assessment was asking of them. This approach ensured that the given measure is comprehended correctly by the students. This nine-item measure was then used in the baseline survey and demonstrated low internal reliability (Cronbach alpha<0.60 [11]).

Based on poor performance, the team undertook the same process again to expand the items on gender attitudes and stereotypes, and dropping those items with very low variability, indicated by >90% in a given cell. A new measure of 15 survey items was constructed and implemented at follow-up, which expanded the items under the areas of gender equality in education and employment, gender roles and female autonomy, to improve the quality of the measure. Two vignettes were used to assess gender attitudes and stereotypes related to education and employment, respectively, with an item added for assessment using the education vignette and two items added for assessment using the employment vignette. Given resource and time constraints, it was not possible to administer both vignettes to all students. Therefore, each vignette was randomly assigned to a student such that a given student received either one vignette or the other. This resulted in N = 3271 students receiving the education vignette and N = 3186 students receiving the employment vignette at follow-up. Current analyses are based on this expanded measure, in two versions, one with the education vignette items and the other with the employment vignette items.

### Measurement

As noted above, participants received a behavioral assessment that included survey items on key demographics, gender attitudes and stereotypes survey items and vignettes, and an IAT.

### Implicit Association Tests

#### Gender attitudes and stereotypes IATs

IAT is a complex measure that requires participants to sort stimulus items simultaneously, i.e., pictures or text, into two response options. For this study, we developed two unique but related IATs, as noted above; these were the Gender-Based Values IAT (IAT1) and the Gender Stereotypes IAT (IAT2). For IAT1 on gender-based value, participants sorted stimuli as good versus bad, indicating attitudes of gender bias in valuing women/girls relative to men/boys. For IAT2 on gender stereotypes, participants sorted stimuli in labor tasks—domestic or traditionally female work versus otherwise.

To ensure accurate data capture and reduce subject confusion or error, the IATs include practice tasks to familiarize respondents with the stimulus materials and sorting rules. Hence, the IAT is provided in seven blocks (Table 1). Blocks after the practice block introduce greater complexity. In the initial block B1, participants practice basic keyboard use and sorting using a less complex concept on which to sort—insects versus flowers. In the first block B2, participants just sort stimuli as boy versus girls. In the second block B3, participants sort the response options as good words or bad words. In blocks B4 and B5, sorting becomes more complex, with items representing boys and good (for example, boys’ faces and good words) receive one response, and items representing girls and bad (in this example, girls’ faces and bad words) receive the alternative response. In blocks B7 and B8, items representing girls and good are sorted with one response, and items representing boys and bad are sorted with the alternative response. During each block, if subjects assign stimuli to wrong groups, a red “X” appears, and subjects press on the correct keys to see the next stimuli. The key assumption here is that subjects who possess stronger associations of positive evaluation with boys compared to girls will find the first sorting task much easier than the second. Likewise, subjects possessing stronger associations of positive evaluation with girls compared to boys might find the second sorting task easier than the first (To control for sequence effects from the order in which images and words are grouped, even numbered subjects got girl/good on one side and boy/bad on the other side as the first sorting task (B1, B3 and B4), and the reverse in the second sorting task (B5, B6 and B7)).

**Table 1:** Typical IAT structure.

Block	Number of Trials	Items assigned to left-key response	Items assigned to right-key response
B1	20	Pictures of flowers	Pictures of insects
B2	20	Faces of boys	Faces of girls
B3	20	Good words	Bad words
B4	20	Faces of boys + Good words	Faces of girls + Bad words
B5	40	Faces of boys + Good words	Faces of girls + Bad words
B6	20	Faces of girls	Faces of boys
B7	20	Faces of girls + Good words	Faces of boys + Bad words
B8	40	Faces of girls + Good words	Faces of boys + Bad words

Ease of sorting is indexed both by the speed of responses as well as the frequency of errors with both faster responses and fewer errors indicating stronger associations. The time elapsed between each stimulus’ appearance and pressing a correct response key is recorded as response latency. In the IATs we administered (structure shown in Tables 2 and 3), the stimuli were randomly drawn with replacements, and in blocks B4, B6, B8 and B10, words and images alternately appeared. While latency for all trials were recorded (except for instruction pages), we followed the standard protocol for IAT and only use latency from blocks B4, B6, B8 and B10 to measure implicit association. The other trials are included for familiarization and practice with which images and words appear in our IAT and what their correct response keys are. IAT 2 follows a similar structure (Table 3) although the stimuli are changed to association of images of men and women with gender stereotyped jobs and roles. S1-S6 Figs in S2 Appendix show example screenshots of IAT1 and IAT2.

**Table 2:** IAT structure for IAT1.

Block	Number of Trials	Description
B1	20	Instructions with practice trials using pictures of flowers and insects
B2	20	Categorizing boy’s faces and girl’s faces
B3	20	Categorizing good words and bad words
B4	20	Categorizing boys and good words into one group, and girls and bad words into another (compatible practice)
B6	40	Same as block 4 (compatible test)
B7	20	Categorizing boy’s faces and girl’s faces to reversed keys
B8	20	Categorizing girls and good words into one group, and boys and bad words into another (incompatible practice)
B10	40	Same as block 8 (incompatible test)

Notes: Block 5 and 9 are one-page instructions given before starting next blocks.

**Table 3:** IAT structure for IAT2.

Block	Number of Trials	Description
B1	20	Instructions with practice trials using pictures of flowers and insects
B2	20	Categorizing men’s faces and women’s faces
B3	20	Categorizing professional tasks and household tasks
B4	20	Categorizing men and professional tasks into one group, and women and household tasks into another (compatible practice)
B6	40	Same as block 4 (compatible test)
B7	20	Categorizing men’s faces and women’s faces to reversed keys
B8	20	Categorizing women and professional tasks into one group, and men and household tasks into another (incompatible practice)
B10	40	Same as block 8 (incompatible test)

Notes: Block 5 and 9 are one-page instructions given before starting next blocks.

Following Lane et al. (2007), we deleted extreme outlier trials where response latencies were greater than 10,000 milliseconds (msec), and deleted subjects for whom more than 10% of trials have latency less than 300 msec [34]. To measure the strength of implicit association, we calculated mean response latencies for blocks girl+good; girl+bad; boy+good; boy+bad for IAT1, and for blocks domestic task+women; domestic task+men; professional task+women; professional task+men for IAT2. As noted above, this was only done for Blocks 4, 6, 8, and 10. We computed an “inclusive” standard deviation for all trials in compatible and incompatible blocks. In order to reduce any influence that the order of pairing might have on the response latencies, we counterbalance the order of pairing for half of the students by presenting the incompatible blocks before the compatible blocks. We used the mean latency from each of the blocks (two compatible and two incompatible) to compute mean differences (Incompatible 1 –Compatible 1) and (Incompatible 2 –Compatible 2). These were then divided by their associated “inclusive” standard deviation; we then calculated the D-measure as the equal-weight average of the two resulting ratios. A higher D-measure, with the specified range of -1 and 1, represents greater implicit preference for girls over boys. The D measure for IAT1 indicates that adolescent girls have higher implicit preference for girls compared to their male peers. On the other hand, IAT2 indicates a slight preference for girls in the sample of adolescents for boys and girls (Table 4). We also calculated mean accuracy and latency for all four blocks in IAT1 and IAT2 (Table 4). Note that despite trimming outliers, the mean latency is higher compared to other studies, such as average latency of 929 msec in Greenwald’s IAT using Bush/Al Gore images and pleasant/unpleasant words, which used an adult sample and did not trim outliers [26]. Feasibility of further improvement in response latencies is uncertain given the sub-population of young adolescents in low technology and low literacy contexts. In the future, other researchers working in similar contexts could consider additional practice tests for respondents to build more comfort with the laptop and the IAT.

**Table 4:** Descriptive statistics on gender attitudes from the IAT.

	*Good versus bad* IAT		*Occupation* IAT	
	Male	Female	Male	Female
Number of students	1,340	1,738	1,489	1,891
Mean latency of first compatible test	1639.77	1850.16	1902.84	1882.31
Mean latency of second compatible test	1359.47	1510.34	1556.92	1553.11
Mean latency of first incompatible test	1802.80	1584.76	1817.27	1831.02
Mean latency of second incompatible test	1467.69	1322.24	1464.28	1470.44
Mean accuracy of first compatible test	0.80	0.72	0.71	0.70
Mean accuracy of second compatible test	0.81	0.74	0.76	0.75
Mean accuracy of first incompatible test	0.76	0.81	0.81	0.80
Mean accuracy of second incompatible test	0.78	0.81	0.82	0.81
Implicit preference for girls	-0.14	0.23	0.10	0.08
Student’s age	14.98	14.72	15.04	14.72
Hindu	0.96	0.96	0.96	0.94
Scheduled Caste	0.32	0.34	0.33	0.31

Compatible tests include the combination ‘boy+good’ and ‘girl+bad’ for the Good versus Bad IAT and ‘boy+professional tasks’ and ‘girl+domestic tasks’ for the Occupation IAT. Incompatible tests include the combination ‘boy+bad’ and ‘girl+good’ for the Good versus Bad IAT and ‘boy+domestic tasks’ and ‘girl+professional tasks’ for the Occupation IAT. The compatible and incompatible tests had two blocks each in the Implicit Association Test.

#### Gender-based value and stereotypes survey and vignette measure

The *Gender-Based Value and Stereotypes Measure* is an 18-item measure inclusive of 15 survey items and 3 additional items which used vignettes. The 15 survey items were on gender-based value and stereotypes in the following areas: gender bias against females/advantaging males in education (2 items), gender bias against females/advantaging males in employment (3 items), gender roles/expectations and female autonomy (9 items), son preference as indicated by fertility preferences (1 item). The two vignettes included a vignette focused on gender bias versus equality in education and a second vignette focused on gender bias versus equality in employment. One item was then used to assess gender bias using the education vignette and two items were used for the employment vignette. For 11 of the 15 survey items and both of the employment vignette items, participants asked how much they agree or disagree on each item using a five-point Likert scale. Four items on gender-based value and stereotypes related to gender bias in employment were based on constructing the differential in a given behavior or attribute ascribed to girls/women and then to boys/men. The item used for the education vignette directly assessed female or male preference for an educational opportunity. Half the participants received the education vignette and corresponding item, and half received the employment vignette and corresponding items. Missing values are imputed by taking the district-gender-treatment average. All items were scored and summated to yield an overall measurement score per Anderson (2008) [35], with a higher score indicating more gender equal attitudes and stereotypes. Details on items and scoring can be seen in S3 Appendix and have been described in Dhar et al. (2022) [11].

## 3. Descriptive analysis—Gender attitudes

We report two sets of descriptive analyses on gender attitudes from the survey and vignette measure, as well as the IAT. The first, represented by the D measure, reports the results on implicit preference for girls for IAT1 and IAT2 (Table 4). The main finding is that for IAT1, which looks at sexist gender attitudes, we find that boys display an implicit negative preference for girls given that their D-measure is negative (-0.14). It is striking that boys disproportionately associate the opposite sex with negative attributes. The results are reversed for girls. Girls are not gender neutral, but disproportionately associate female with good attributes with a positive D measure of 0.23. For IAT2, which measures stereotypes of gender roles, both boys and girls surprisingly display a slight positive implicit preference for girls, indicating that IAT2 does not show strong gender stereotypes related to household work and employment outside the house in this sample.

The second, the Gender Attitude Index from the Gender-Based Value and Stereotype Survey and Vignette Measure in Table 5 shows that girls tend to be more progressive than boys on their gender attitudes, with the overall score -0.07 for boys and 0.05 for girls. The difference is visible in certain survey items. For example, 68% of girls disagree that a man should have the final word about decisions in the home, as opposed to 46% for boys. Almost half of the girls disagree that boys should get more resources and opportunities for education than girls, whereas less than a third of boys disagree with that statement.

**Table 5:** Descriptive statistics on gender attitudes from survey and vignette measure.

	Male	Female
Number of Observations	2,829	3,629
Disagree: Wives should be less educated than their husbands.	0.67	0.80
Agree: It would be a good idea to elect a woman as the Sarpanch of your village.	0.89	0.94
Disagree: Boys should get more opportunities and resources for education	0.30	0.46
Would have sent Rakhi or borrowed money to send both for further studies	0.68	0.89
Disagree: A man should have the final word about decisions in his home.	0.46	0.68
Disagree: A woman should tolerate violence in order to keep her family together.	0.75	0.85
Disagree: Parents should maintain stricter control over daughters than sons	0.36	0.53
Disagree: Woman’s most important role is take care of home, feed kids and cook	0.40	0.62
Disagree: Men are better suited than women to work outside the house	0.41	0.66
Agree: Daughters should have a similar right to inherited property as sons.	0.92	0.94
% say boy should be demure/% say girl should be demure	0.98	0.87
% say boy cover mouth when laughing/% say girl cover mouth when laughing	0.81	0.81
Disagree: Marriage is more important for Pooja than her job	0.54	0.65
Disagree: Being a teacher would be more suitable for Pooja	0.44	0.50
Sister/cousins/female friends should be married after age 19	0.63	0.78
Difference between boys and girls age to marry is lesser than control median	0.56	0.62
Girls should attain higher education less Boys should attain higher education	-0.09	0.02
Agree: Women should be allowed to work outside home	0.61	0.90
Agree to have no more children after two girls, but not after two boys	0.89	0.93
Gender Attitudes Index	-0.07	0.05
Student’s age	15.01	14.72
Hindu	0.96	0.95
Scheduled caste	0.33	0.32

## 4. Empirical analysis—Psychometric properties

In this section, we check for the robustness of the IAT measures and test its psychometric properties, in comparison to the gender-based value and stereotypes survey and vignette-based measure.

### Internal consistency

Internal consistencies are measured by how homogeneous the responses are to all items in a particular measure [36]. Current conventions suggest that inter-item consistencies of 0.80 (20% error) or higher are acceptable [34], although many widely used scales remain around 0.70 [37]. Previous studies on the IAT have found that implicit attitude measures generally have low inter-item consistency and typically do not fare as well as self-reported measures in this regard. Table 6 reports inter-item consistency for the IATs by calculating Cronbach’s alpha using the response latency for compatible and incompatible blocks [36]. We find that IAT1 and IAT2 have comparable consistencies, with Cronbach alphas of 0.72 for IAT1 and 0.61 for IAT2.

**Table 6:** Internal consistency (Cronbach alpha) of IATs.

	Cronbach Alpha
Self-reported Indices	
Gender Attitudes Index	0.7228
Attitudes towards Female Gender Roles Sub- Index	0.7442
Age of Marriage Sub-Index	0.5389
Social and Economic Empowerment Sub-Index	0.2232
Higher Education and Employment Sub-Index	0.2430
Attitudes towards Fertility Sub-Index	0.0448
Gendered Behavior Sub-Index	0.0973
Implicit Measures	
Good versus Bad IAT	0.7237
Occupation IAT	0.6091

We compare the internal consistency of the IATs with the gender attitude index and find that the IAT1 is as consistent as the gender attitude index (*a* = 0.72), but this is not true for IAT2.

### Validity

Using guidance from applied psychology, validity testing is designed to assess whether the measure is in fact measuring the construct of focus by assessing its association with the same construct if a gold standard measure of that construct is available (criterion validity), a similar construct if no gold standard measure exists (criterion-related validity), or a variable or outcome one would theorize to be associated with the measure if it was actually measuring the construct of focus (construct validity) [38]. To assess criterion-related validity, we compared IAT scores to the gender attitude index, as both measures assess a similar construct and there is no gold standard measure of gender attitudes in the field. We use the following equation to estimate the correlation between IAT responses and the gender attitude index.

$${\text{Y}}_{\text{ij}}=\beta_{\text{o}}+\beta_{1}{\text{X}}_{\text{ij}}+\gamma_{\text{gd}}+\Phi_{\text{gc}}+\varepsilon_{\text{ij}}$$ (1)

where Y_ij_ is the outcome of interest (IAT) for student i in school j. X_ij_ is the gender attitude index and γ_gd_ and Φ_gc_ denote district-gender and gender-grade fixed effects respectively. We also run a specification without controls.

Table 7 indicates that IAT1 shows a modest and significant correlation of 0.440 (p<0.01) with the gender attitude index. However, the correlation weakens with the inclusion of gender-grade and district-gender fixed effects in Column 2 (0.089, p<0.05). Relative to IAT1, IAT2 shows a weaker correlation with the gender attitude index without fixed effects (0.118, p<0.05), but a stronger correlation when controlling for fixed effects (0.156, p<0.01). Overall, the results suggest that positive implicit preference captured in the IATs corresponds to more progressive responses in the gender index, and there is a helpful correlation between explicitly progressive views and implicit preference for girls.

**Table 7:** Criterion-related validity.

	Implicit preference or girls (Good versus bad)	Implicit preference or girls (Good versus bad)	Implicit preference or girls (Occupation)	Implicit preference or girls (Occupation)
	(1)	(2)	(3)	(4)
Gender Attitudes Index	0.440***	0.089**	0.118**	0.156***
	[0.044]	[0.044]	[0.049]	[0.054]
Basic controls	No	Yes	No	Yes
Observations	2926	2926	3198	3198

Notes: Basic controls include gender-grade and district-gender fixed effects, and standard errors are clustered at the school level.

* p<0.10,

**p<0.05,

***p<0.01.

We also test the construct validity of the IAT by examining whether IAT correlates with girls’ individual behavioral outcomes (such as mobility) and educational achievement (attendance in school, grade completion), given that we would anticipate that girls with more traditional gender attitudes would report more restrictive or poorer behavioral outcomes. We estimate the correlation between IAT responses and self-reported measures of gender related behavior using the following model.

$${\text{Y}}_{\text{ij}}=\beta_{\text{o}}+\beta_{1}{\text{X}}_{\text{ij}}+\gamma_{\text{gd}}+\Phi_{\text{gc}}+\varepsilon_{\text{ij}}$$ (2)

where Y_ij_ is the outcome of interest (IAT score for IAT1 or IAT2) for student i in school j. X_ij_ is the measure of girls’ agency such as mobility or school enrolment. Among boys, X_ij_ is based on boys’ willingness to intervene when a girl at school is being teased, their contribution to household work, or discouragement for sisters and girl cousins to meet friends. As before, γ_gd_ and Φ_gc_ are district-gender and gender-grade fixed effects, respectively. We run specifications with and without controls.

Table 8 reports that both IAT scores are modestly and significantly correlated with girls’ mobility (0.128, p<0.01 for IAT1 in Column 1; 0.092, p<0.05 for IAT2 in Column 6) as well as with girls’ school enrolment (0.108, p<0.01 in Column 2 and 0.139, p<0.05 in Column 7). For boys, we find that IAT2, but not IAT1, is significantly correlated with their intervention if a girl was being teased in school (0.108, p<0.05 in Column 10). IATs were not correlated with other behaviors for boys, such as household work or encouragement for your sister or cousin to meet friends. These results persist with the inclusion of controls (available on request). These correlations suggest that the IATs have some ability to predict variables of self-reported gender-related behavior.

**Table 8:** Construct validity and correlation with behavioral outcomes.

	Implicit preference or girls (Good vs bad)	Implicit preference or girls (Good vs bad)	Implicit preference or girls (Good vs bad)	Implicit preference or girls (Good vs bad)	Implicit preference or girls (Good vs bad)	Implicit preference or girls (Occupation)	Implicit preference or girls (Occupation)	Implicit preference or girls (Occupation)	Implicit preference or girls (Occupation)	Implicit preference or girls (Occupation)
	(1)	(2)	(3)	(4)	(5)	(6)	(7)	(8)	(9)	(10)
Are you allowed to go school alone or with friends? (Girls only)	0.128***					0.092**				
	[0.036]					[0.044]				
Child is enrolled in school at second round (Girls only)		0.108***					0.139**			
		[0.041]					[0.054]			
Child cooks/cleans/washes clothes atleast once a week (Boys only)			0.018					-0.028		
			[0.021]					[0.027]		
Do you discourage your sister/female cousin to meet her friends? (Boys only)				0.029					0.046	
				[0.045]					[0.046]	
Have you intervened if a girl was being teased in your school? (Boys only)					-0.026					0.108**
					[0.036]					[0.047]
Basic controls	No	No	No	No	No	No	No	No	No	No
Observations	1625	1676	1250	1249	393	1774	1826	1372	1371	431

Notes:

* p<0.10,

**p<0.05,

***p<0.01.

## 5. Discussion

Measuring gender attitudes well has been a continuing challenge, especially for adolescents in low-income contexts, underlining the need to reassess and improve measurement of gender attitudes. Better measures are needed, and vignettes and IATs in particular offer a potentially valuable addition to the mix of existing instruments to capture gender attitudes that can circumvent dilemmas such as false reporting and social desirability bias, which can hinder reliable measurement of gender attitudes.

This paper extends our understanding of effective approaches of measuring gender attitudes by assessing the psychometric properties of two novel IATs customized for adolescent boys and girls in India, as well as a survey assessment inclusive of vignettes. The inclusion of both allows for benchmarking the performance of the IATs with a gender attitudes index comprised of responses from self-reported survey questions and vignettes, allowing for a criterion-related validity assessment in the absence of a gold standard measure of gender attitudes in this population. To our knowledge, these are the first IAT scales to measure gender attitudes among adolescents in India. IAT1 focuses on general positive and negative attitudes associated with gender, and the second (IAT2) measures attitudes associated with gender-stereotypical employment. These IATs can serve as prototypes for those interested in measuring gender attitudes with adolescents in India or other similar contexts. Importantly, our measures all demonstrated good internal reliability and validity.

IATs can be a useful technique to measure gender attitudes and discrimination among different sub-populations, and the concepts/associations can be adapted based on the needs of the project. The IATs capture interesting results which offer insights into the implicit gender attitudes of youth in India. The gender-values focused IAT1 results signal that male students hold a negative implicit preference for girls, in contrast to their female peers who display a strong positive preference for girls. This pattern is similar to the gender attitudes index, comprised of items from survey questions and vignettes, which shows that female students have more progressive views than do their male counterparts. IAT2, however, shows that both male and female students do not hold strong implicit stereotypes on gender roles within and outside the household, diverging from their explicit views in self-reported questions and vignettes where there is stronger stereotyping.

Despite being administered in a low digital literacy context with a low-income population, we find that the gender IATs are consistent, valid, and moderately correlated with outcomes. Our results show that IATs such as IAT1 can perform just as well as direct survey questions in terms of consistency. IATs display a modest correlation with survey and vignette based measures of gender attitudes and also have some predictive power for outcome variables for individual gender-related behavior and outcomes such as girls’ mobility and school enrolment or boys’ intervention when a girl is being teased. These findings suggest that despite the cost and resource intensity of customized IATs, they may provide a useful addition and unbiased alternative to survey-based measures relying on direct questions. While the current technology and time to administer IATs makes them prohibitive to incorporate in at-scale surveys, they expand the mix of novel tools to measure certain biases and stereotypes.

The limitations of this study include the fact that we rely only on self-reported measures for behavioral outcomes used to measure the criterion validity of the IAT. Ideally, there would be a gold standard to measure gender attitudes for adolescents in India, but given there were no such available measures, we benchmark the IAT with a customized direct survey and vignette based measure developed specifically for the study (i.e., testing criterion-related validity). Since the implementation of the study, other measures on gender attitudes and norms have been released for use with adolescents in India (for instance, Blum et al’s (2019) Global Early Adolescent Study [28]), providing the opportunity to do more robust validity testing of the IAT using these new measures. Further, the measures were only tested with school students in a single state in India, potentially limiting their generalizability to other contexts and sub-populations. Lastly, an important limitation is that we use data from a follow-up survey of an existing evaluation study, which had not been explicitly designed to test these measures. Measures such as the IAT and vignette were randomized in their administration to individuals, so we did not leverage the entire sample of respondents—however, the samples were large enough and the imputation was done in a technically sound manner.

Our study introduces a new IAT based instrument to measure adolescent gender attitudes in India and contributes to the set of existing tools for measuring and investigating gender attitudes in developing countries and among adolescents. The IAT tools and the design strategy can be used for further customization or for creating other similar IATs to systematize response latency measures for gender attitude measurement. Further refinements should be considered such as additional computer-based practice rounds for respondents to get more comfortable with the test or a complete switch away from words to images to simplify the test further for a lower literacy population. Nevertheless, we hope that this effort at improving IATs will stimulate further research and efforts in this direction to improve IAT testing and come up with other innovative creative approaches to measuring gender attitudes. Given that the tool is less prone to social desirability bias compared to traditional methods, we also hope that our design strategy can facilitate more developing country research based on IATs in domains such as education, transition to work, domestic violence and women’s agency (See Purkayastha et al. (2003) [39] for a review of topics).

## Supporting information

S1 Appendix (DOCX)

S2 Appendix (DOCX)

S3 Appendix (DOCX)
