# Supplementary material for: Measuring gender attitudes: Developing and testing Implicit Association Tests for adolescents in India
Source: PLoS One. 2022 Jun 16;17(6):e0264077. doi: 10.1371/journal.pone.0264077 (PMC9202834; doi:10.1371/journal.pone.0264077)
Supplement: S1 Appendix — (DOCX) [file pone.0264077.s001.docx]

**Appendix Table S1: Sample characteristics by IAT and gender**

|  | *Good versus bad* IAT | | *Occupation* IAT | |
| --- | --- | --- | --- | --- |
|  | Male | Female | Male | Female |
| Number of students | 1,340 | 1,738 | 1,489 | 1,891 |
| Student’s age | 14.98 | 14.72 | 15.04 | 14.72 |
| Hindu | 0.96 | 0.96 | 0.96 | 0.94 |
| 6th Grade | 0.51 | 0.57 | 0.49 | 0.58 |
| Scheduled caste | 0.32 | 0.34 | 0.33 | 0.31 |
| Sonipat | 0.29 | 0. 29 | 0.33 | 0.35 |
| Panipat | 0.21 | 0.21 | 0.23 | 0.22 |
| Rohtak | 0.33 | 0.35 | 0.27 | 0.29 |
| Mother’s age | 35.11 | 35.61 | 35.24 | 35.31 |
| Father’s age | 40.71 | 40.28 | 40.88 | 39.93 |
| Mother is illiterate | 0.36 | 0.38 | 0.40 | 0.37 |
| Mother works full-time | 0.13 | 0.18 | 0.12 | 0.17 |
| Dwelling has flush toilet | 0.12 | 0.17 | 0.11 | 0.17 |

**Appendix Table S2: Attrition**

| Variable | Attrited | Non-attrited | P-value | Std. diff. |
| --- | --- | --- | --- | --- |
| Number of students | 1,894 | 6,458 |  |  |
| Student’s age | 11.752  (1.226) | 12.008  (1.135) | 0.000 | 0.204 |
| Hindu | 0.954  (0.209) | 0.937  (0.244) | 0.002 | -0.078 |
| 6th Grade | 0.541  (0.498) | 0.518  (0.500) | 0.083 | -0.046 |
| Scheduled caste | 0.325  (0.468) | 0.294  (0.456) | 0.012 | -0.067 |
| Sonipat | 0.313  (0.464) | 0. 428  (0.495) | 0.000 | 0.243 |
| Panipat | 0.218  (0.413) | 0.389  (0.488) | 0.000 | 0.391 |
| Rohtak | 0.310  (0.462) | 0.074  (0.263) | 0.000 | -0.540 |
| Mother’s age | 35.335  (6.150) | 35.689  (6.940) | 0.232 | 0.056 |
| Father’s age | 40.418  (6.964) | 41.136  (7.524) | 0.063 | 0.101 |
| Mother is illiterate | 0.379  (0.485) | 0.436  (0.496) | 0.011 | 0.117 |
| Mother works full-time | 0.154  (0.361) | 0.114  (0.318) | 0.015 | -0.114 |
| Dwelling has flush toilet | 0.147  (0.354) | 0.139  (0.346) | 0.369 | -0.023 |
| Implicit preference for girls | 0.034  (0.398) | 0.043  (0.387) | 0.421 | 0.023 |

**Appendix S3: Construct validity and correlation with behavioral outcomes, with controls**

|  | Implicit preference or girls (Good vs bad)  (1) | Implicit preference or girls (Good vs bad)  (2) | Implicit preference or girls (Good vs bad)  (3) | Implicit preference or girls (Good vs bad)  (4) | Implicit preference or girls (Good vs bad)  (5) | Implicit preference or girls (Occupation)  (6) | Implicit preference or girls (Occupation)  (7) | Implicit preference or girls (Occupation)  (8) | Implicit preference or girls (Occupation)  (9) | Implicit preference or girls (Occupation)  (10) |
| --- | --- | --- | --- | --- | --- | --- | --- | --- | --- | --- |
| Are you allowed to go school alone or with friends? (Girls only) | 0.129∗∗∗  [0.037] |  |  |  |  | 0.101∗∗  [0.041] |  |  |  |  |
| Child is enrolled in school at second round (Girls only) |  | 0.114∗∗∗  [0.037] |  |  |  |  | 0.143∗∗  [0.060] |  |  |  |
| Child cooks/cleans/washes clothes atleast once a week (Boys only) |  |  | 0.022  [0.022] |  |  |  |  | -0.025  [0.027] |  |  |
| Do you discourage your sister/female cousin to meet her friends? (Boys only) |  |  |  | 0.019  [0.043] |  |  |  |  | 0.031  [0.044] |  |
| Have you intervened if a girl was being teased in your school? (Boys only) |  |  |  |  | -0.021  [0.036] |  |  |  |  | 0.108∗∗  [0.051] |
| Basic controls | Yes | Yes | Yes | Yes | Yes | Yes | Yes | Yes | Yes | Yes |
| Observations | 1625 | 1676 | 1250 | 1249 | 393 | 1774 | 1826 | 1372 | 1371 | 431 |

Notes: Basic controls include gender-grade and district-gender fixed effects, and standard errors are clustered at the school level. * p<0.10, **p<0.05, ***p<0.01.
