# Supplementary material for: Measuring gender attitudes: Developing and testing Implicit Association Tests for adolescents in India
Source: PLoS One. 2022 Jun 16;17(6):e0264077. doi: 10.1371/journal.pone.0264077 (PMC9202834; doi:10.1371/journal.pone.0264077)
Supplement: S2 Appendix — (DOCX) [file pone.0264077.s002.docx]

**Appendix 2: Implicit Association Tests**

**Figure S1: IAT 1 Block 2 and 3**





Note: Images used under creative commons license.

**Figure S2: IAT1 Block 4 and 6**


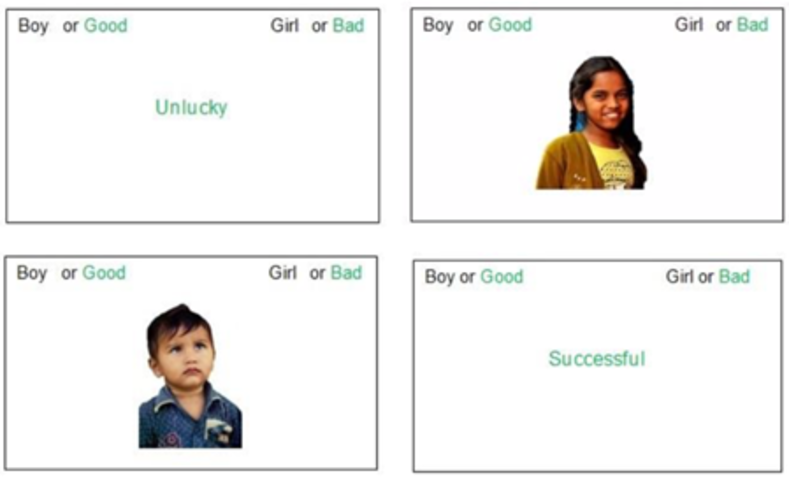


Note: Images used under creative commons license.

**Figure S3: IAT1 Block 7 and 8**


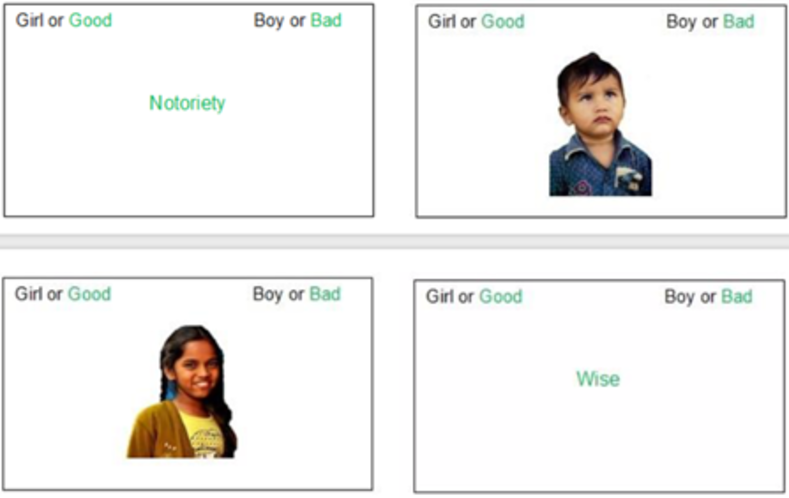


Note: Images used under creative commons license.

**Figure S4: IAT2 Block 3 and 4**


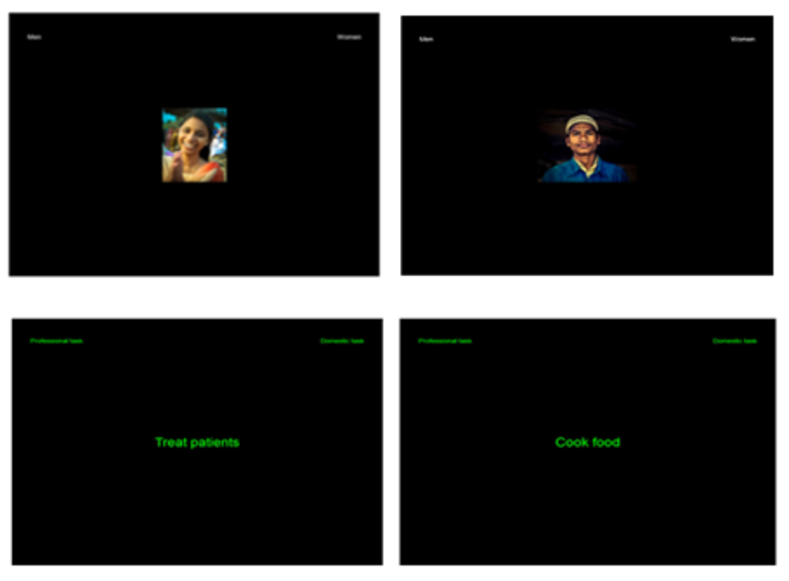


Note: Images used under creative commons license.

**Figure S5: IAT2 Block 4 and 6**


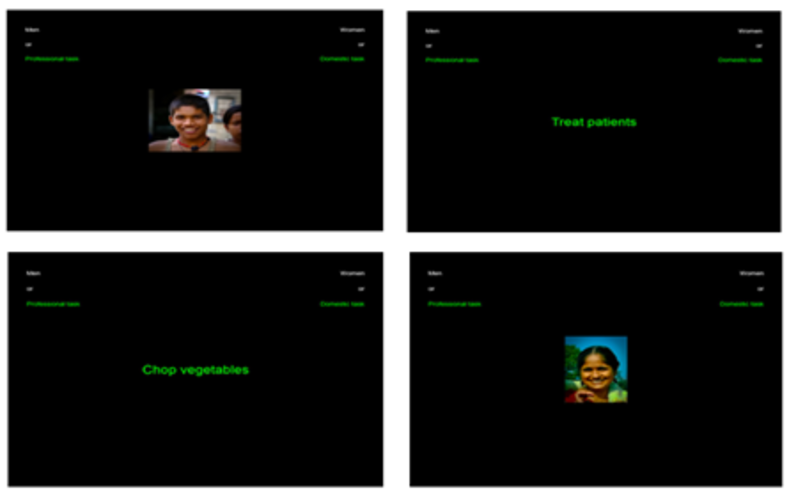


Note: Images used under creative commons license.

**Figure S6: IAT2 Block 7 and 8**


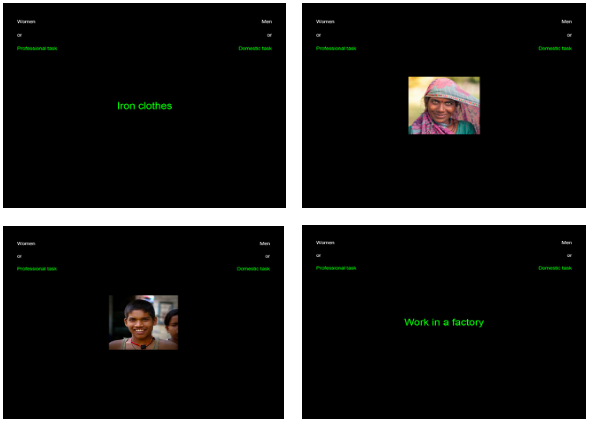


Note: Images used under creative commons license.
