# Supplementary material for: Measuring gender attitudes: Developing and testing Implicit Association Tests for adolescents in India
Source: PLoS One. 2022 Jun 16;17(6):e0264077. doi: 10.1371/journal.pone.0264077 (PMC9202834; doi:10.1371/journal.pone.0264077)
Supplement: S3 Appendix — (DOCX) [file pone.0264077.s003.docx]

**Appendix 3: Gender Attitudes and Stereotypes Survey Items and Vignettes**

The Gender Attitudes and Stereotypes Measure is an 18-item measure inclusive of 15 survey items and 3 additional items which used vignettes. The 15 survey items were on gender attitudes and stereotypes in the following areas:

- gender bias against females/advantaging males in education (2 items),
- gender bias against females/advantaging males in employment (3 items)
- gender roles/expectations and female autonomy (9 items)
- son preference (1 item)

The two vignettes included a vignette focused on gender bias versus equality in education and a second vignette focused on gender bias versus equality in employment. One item was then used to assess gender bias using the education vignette and two items were used for the employment vignette. All individual items and their response patterns are indicated below, and the index is the weighted average value of the individual items, with weights constructed by normalizing the variables to have the same standard deviation and then recovering the weights given by the inverse covariance matrix [35]. Asterix (*) on the question indicates that the variable is included in the overall gender attitudes index.

The following steps were involved in producing the final indices:

1. The individual variables are first converted to dummy variables. For questions that used a 5-point Likert scale, the binary variable was coded as 1 if the respondent answered “Strongly Agree” or “Agree” with a gender-progressive statement (or “Strongly Disagree” or “Disagree” with a gender-regressive statement), and 0 otherwise
2. We impute missing values with gender-district-treatment averages.
3. Following Anderson (2008) and Dhar et al (2022), the outcome index is created using variance-weighted averages. Weights are based on the control group [16,35].
4. This weighted index is then re-scaled such that the control group mean is 0 and the standard deviation is 1.

|  | **Individual item** | **Response item** | **Variable construction** |
| --- | --- | --- | --- |
| **A.** | **Attitudes towards Female Gender Roles** | |  |
| 1. | Wives should be less educated than their husbands* | 1 – Strongly agree  2 – Agree  3 – Neither Agree nor disagree  4 – Disagree  5 – Strongly disagree  -999-Don’t Know  -998-Refuse to answer | The variable is coded as gender if the respondent answered “Strongly Disagree” or “Disagree”, and 0 otherwise |
| 2. | Boys should be allowed to get more opportunities and resources for education* | 1 – Strongly agree  2 – Agree  3 – Neither Agree nor disagree  4 – Disagree  5 – Strongly disagree  -999-Don’t Know  -998-Refuse to answer | The variable is coded as 1 if the respondent answered “Strongly Disagree” or “Disagree”, and 0 otherwise |
| 3. | A man should have the final word about decisions in his home* | 1 – Strongly agree  2 – Agree  3 – Neither Agree nor disagree  4 – Disagree  5 – Strongly disagree  -999-Don’t Know  -998-Refuse to answer | The variable is coded as 1 if the respondent answered “Strongly Disagree” or “Disagree”, and 0 otherwise |
| 4. | Parents should maintain stricter control over their daughters than their sons* | 1 – Strongly agree  2 – Agree  3 – Neither Agree nor disagree  4 – Disagree  5 – Strongly disagree  -999-Don’t Know  -998-Refuse to answer | The variable is coded as 1 if the respondent answered “Strongly Disagree” or “Disagree”, and 0 otherwise |
| 5. | A woman’s most important role is to take care of her home, feeding kids and cook for her family* | 1 – Strongly agree  2 – Agree  3 – Neither Agree nor disagree  4 – Disagree  5 – Strongly disagree  -999-Don’t Know  -998-Refuse to answer | The variable is coded as 1 if the respondent answered “Strongly Disagree” or “Disagree”, and 0 otherwise |
| 6. | Men are better suited than women to work outside the house* | 1 – Strongly agree  2 – Agree  3 – Neither Agree nor disagree  4 – Disagree  5 – Strongly disagree  -999-Don’t Know  -998-Refuse to answer | The variable is coded as 1 if the respondent answered “Strongly Disagree” or “Disagree”, and 0 otherwise |
| **B.** | **Age of Marriage** | |  |
| 7. | At what age would you like your sister/female cousins/friends to get married *minus* At what age would you like your brother/male cousins/friends to get married?* | [integer] | The variable is coded as gender-progressive if age for girls > 19 |
| 8. | At what age would you like your sister/female cousins/friends to get married *minus* At what age would you like your brother/male cousins/friends to get married?* | [integer] | The variable is coded as gender-progressive if the gap between boys and girls is larger than the control group median response |
| **C.** | **Social and Economic Empowerment** | |  |
| 9. | Daughters should have a similar right to inherited property as sons* | 1 – Strongly agree  2 – Agree  3 – Neither Agree nor disagree  4 – Disagree  5 – Strongly disagree  -999-Don’t Know  -998-Refuse to answer | The variable is coded as 1 if the respondent answered “Strongly Agree” or “Agree”, and 0 otherwise |
| 10. | It would be a good idea to elect a woman as the Sarpanch of your village* | 1 – Strongly agree  2 – Agree  3 – Neither Agree nor disagree  4 – Disagree  5 – Strongly disagree  -999-Don’t know  -998-Refuse to answer | The variable is coded as 1 if the respondent answered “Strongly Agree” or “Agree”, and 0 otherwise |
| **D.** | **Higher education and Employment** | |  |
| 11. | Education Vignette: If you were the head of the family, who would you have sent to the town for further studies?* | 1- Rajat  2- Rakhi  3- Borrowed money and sent both  -999-Don’t know  -998-Refuse to answer | The variable is coded as gender-progressive if the respondent answered ‘Rakhi’ or ‘borrowed money and sent both’ |
| 12. | Work Vignette: Marriage is more important for Pooja than her job* | 1 – Strongly agree  2 – Agree  3 – Neither Agree nor disagree  4 – Disagree  5 – Strongly disagree  -999-Don’t know  -998-Refuse to answer | The variable is coded as 1 if the respondent answered “Strongly Disagree” or “Disagree”, and 0 otherwise |
| 13. | Work Vignette: Being a teacher would be more suitable for Pooja* | 1 – Strongly agree  2 – Agree  3 – Neither Agree nor disagree  4 – Disagree  5 – Strongly disagree  -999-Don’t know  -998-Refuse to answer | The variable is coded as 1 if the respondent answered “Strongly Disagree” or “Disagree”, and 0 otherwise |
| **E.** | **Attitudes towards Fertility** | |  |
| 14. | Girls should attain higher education so that they find better husbands *minus* Boys should attain higher education so that they find better wives * | 1 – Strongly agree  2 – Agree  3 – Neither Agree nor disagree  4 – Disagree  5 – Strongly disagree  -999-Don’t know  -998-Refuse to answer | The variable is coded as the differential between these two questions |
| 15. | Suppose the first two children born to a husband and wife are both girls. Which of the following should they do? *minus* Suppose the first two children born to a husband and wife are both boys. Which of the following should they do?* | 1- Have no more children because they've reached a family size of 2  2- Have one more child, hoping it’s a boy  3- Have more children, until a boy is born  -999-Don’t know  -998-Refuse to answer | The variable is coded as -1 (gender regressive) if the respondent said ’have no more children’ after having  two boys but not after having two girls, 1 (gender progressive) if she said ’have no more children’ after two  girls but not two boys, and 0 otherwise |
| **F.** | **Gendered Behavior** | |  |
| 16. | A shy demeanor makes a boy a more suitable groom *minus* A shy demeanor makes a girl a more suitable bride | 1 – Strongly agree  2 – Agree  3 – Neither Agree nor disagree  4 – Disagree  5 – Strongly disagree  -999-Don’t know  -998-Refuse to answer | Only one of these two questions was asked to each respondent (determined by randomizing). We thus use the school mean rather than the individual’s response. The variable is coded as gender-regressive if the value for demure bride (girl laughs) is greater than a demure groom (boy laughs) |
| 17. | When a girl laughs, she should cover her mouth *minus* When a boy laughs, he should cover his mouth | 1 – Strongly agree  2 – Agree  3 – Neither Agree nor disagree  4 – Disagree  5 – Strongly disagree  -999-Don’t know  -998-Refuse to answer | Only one of these two questions was asked to each respondent (determined by randomizing). We thus use the school mean rather than the individual’s response. The variable is coded as gender-regressive if the value for girl laughs is greater than boy laughs |

**Vignettes**

Gender Equality in Education Vignette. Now we will read you a short description of a family. We will ask you a couple of questions about what you think the father should have done. There are no ‘right’ or ‘wrong’ answers. Please answer each in terms of your own reactions.

*There exists a lower middle class family in a village. The family consists of three children, their parents and grandparents. Among the three children, the two elder ones Rakhi and Rajat are twins. They have a younger brother Ramesh.*

*Rakhi and Rajat have just passed their HSC exams with 80% marks. Both of them have aspirations to go to the nearby town and study in a good college. But that will require them to stay in a hostel in the town independently.*

*The family has got just enough money to send only one of their two children to the town. They also have another younger son to take care of. Finally, their father decides that Rajat should continue his studies whereas Rakhi will stay in the home and help her mother in the household chores and eventually get married.*

Gender Equality in Employment Vignette. Now we will read you a short description of a family. We will ask you a couple of questions about what you think the parents should have done. There are no ‘right’ or ‘wrong’ answers. Please answer each in terms of your own reactions.

*Pooja, a 21-year-old girl belongs to a village in Haryana. Since childhood, she has aspirations of becoming a police officer. After graduating from college, she appears for the Haryana police examination and is offered a job as a police officer.*

*Her parents are worried about her job as they think that is not suitable for a woman. They also believe that it is her age to get married and they have found a prospective groom for her from a good family. Pooja, however, wants to take up the job and does not wish to get married.*

*According to her parents, Pooja would not need to work after she gets married as her husband will take care of her. Pooja should, instead, focus on household work, help out her mother in law and eventually have children. Finally, her parents decide that instead of taking up the job, she should get married.*
